# An improved method for calibrating time-of-flight Laue single-crystal neutron diffractometers

**DOI:** 10.1107/S1600576714006657

**Published:** 2014-05-29

**Authors:** Craig L. Bull, Michael W. Johnson, Hayrullo Hamidov, Kazuki Komatsu, Malcolm Guthrie, Matthias J. Gutmann, John S. Loveday, Richard J. Nelmes

**Affiliations:** a ISIS Facility, STFC Rutherford Appleton Laboratory, Harwell Science and Innovation Campus, Chilton, Didcot, Oxfordshire OX11 OQX, England; b SUPA, School of Physics and Astronomy and Centre for Science at Extreme Conditions, University of Edinburgh, Mayfield Road, Edinburgh EH9 3JZ, Scotland

**Keywords:** neutron diffraction, neutron instruments, time-of-flight, calibration

## Abstract

A robust and comprehensive method for determining the orientation matrix of a single-crystal sample using the neutron Laue time-of-flight (TOF) technique is described. The new method enables the measurement of the unit-cell parameters with an uncertainty in the range 0.015–0.06%, depending upon the crystal symmetry and the number of reflections measured. The improved technique also facilitates the location and integration of weak reflections, which are often more difficult to discern amongst the increased background at higher energies. The technique uses a mathematical model of the relative positions of all the detector pixels of the instrument, together with a methodology that establishes a reproducible reference frame and a method for determining the parameters of the instrument detector model. Since all neutron TOF instruments require precise detector calibration for their effective use, it is possible that the method described here may be of use on other instruments where the detector calibration cannot be determined by other means.

## Abstract 2

An improved method of calibrating neutron time-of-flight single-crystal instruments is described. The calibration method has led to improved lattice parameter determination and ability of the orientation matrix to describe the reflection positions on the detector surface.

## 1. Introduction   

Initially proposed in 1956 by Lowde (Lowde, 1956 ▶), the single-crystal time-of-flight (TOF) Laue technique has now become established as an experimental technique with instruments at many of the world’s existing or former pulsed neutron sources (Schultz, 1987 ▶; Alkire *et al.*, 1985 ▶; Niimura *et al.*, 1983 ▶; Forsyth *et al.*, 1988 ▶; Schultz *et al.*, 1984 ▶; Keen *et al.*, 2006 ▶; J-PARC in Japan, http://j-parc.jp/researcher/MatLife/en/instrumentation/ns_spec.html#bl; SNS in USA, http://neutrons.ornl.gov/topaz).

The spallation method (Carpenter, 1977 ▶) produces pulsed polychromatic beams of neutrons, which fall on the single-crystal sample. A large proportion of a reciprocal lattice row can be scanned by varying the neutron wavelength (as found in one pulse) with a fixed detector. As the neutrons are sorted by velocity and hence wavelength, this allows us to resolve the individual Bragg reflections by TOF. A large three-dimensional portion of reciprocal space is sampled during each neutron pulse and hence many Bragg reflections can be measured simultaneously from a stationary single crystal (given enough detector coverage). The detector and sample need only be moved minimally during data collection, thus simplifying the experimental method. This method has been well described elsewhere (Schultz, 1993 ▶, 1987 ▶; Alkire *et al.*, 1985 ▶; Niimura *et al.*, 1983 ▶; Forsyth *et al.*, 1988 ▶; Schultz *et al.*, 1984 ▶; Keen *et al.*, 2006 ▶). The first single-crystal structure to be solved independently using the TOF Laue technique was published in 1983 and was measured on the SCD instrument at the IPNS (Schultz *et al.*, 1983 ▶).

As the technique uses a white beam and large-area pixel­ated detectors, it is not necessary to know the crystal orientation to measure the Bragg intensities. Having collected sufficient data, it is possible to search the TOF spectrum for reflections using an appropriate search algorithm. The algorithm returns the peak TOF and position on the detector surface, which can then be indexed (given an approximate set of lattice parameters) to determine an orientation matrix (containing information about the crystal’s unit cell and orientation), often by an auto-indexing program. The accuracy of the orientation matrix is dependent on the number of reflections and a knowledge of the instrument and beam geometry relative to the sample (crystal-to-detector distance, detector dimensions and detector angle relative to the incident beam) (Schultz, 1993 ▶).

However, where access to reciprocal space is limited as a result of sample environment, as is the case in high-pressure experiments (Bull, Guthrie, Nelmes, Loveday, Komatsu *et al.*, 2009 ▶) or with the use of cryomagnets, there are limited data for the determination of the unit-cell parameters. Also, such a sample environment increases the background signal in the TOF spectrum, making indexing and integration of weak high-*Q* [*Q* = (4π/λ)sinθ, where 2θ is the scattering angle and λ is the wavelength of the incident radiation] reflections more difficult. In such cases there is a need to improve the accuracy and precision in the determined orientation matrix and integrated intensities.

At the SCD instrument at the IPNS (Argonne, USA), the instrument and beam geometry were determined using a standard test crystal placed at the sample position. The data were collected and the refinement of parameters was performed so as to adjust instrument parameters such as the nominal detector position, orientation and pixel size (Schultz *et al.*, 2006 ▶). Alternatively, these were refined from the data collection in progress and ported to future data collections, provided no changes were made to the detector position (Schultz, 1993 ▶). These methods were implemented using the *ISAW* software suite, which is also used at the new TOPAZ instrument at the SNS (Oak Ridge, USA; Schultz *et al.*, 2006 ▶; Jogl *et al.*, 2011 ▶). In this method, the coordinates of three vectors used to describe the position of each detector and the sample-to-detector distance are refined so as to optimize the indexing of a standard crystal of ruby (Schultz *et al.*, 2006 ▶). On the SXD instrument at the ISIS neutron source (Oxfordshire, UK; Keen *et al.*, 2006 ▶; Gutmann *et al.*, 2003 ▶), the detector positions and geometry are refined for each experiment and each sample orientation, as described in detail later. A similar calibration method is also implemented on the iBIX single-crystal instrument at the J-PARC neutron facility in Japan (http://j-parc.jp/researcher/MatLife/en/instrumentation/ns_spec.html#bl) where, for each of the detectors, three translations and two rotations are used to refine the detector parameters for each sample orientation.

The Laue TOF method has historically provided reduced accuracy in the determination of lattice parameters compared with that provided by monochromatic single-crystal methods, and hence gives an orientation matrix that does not accurately describe the crystal orientation relative to the instrument. This does not mean that the indexing of reflections is incorrect, but it does have implications for the quality or reliability of the data collected (for accuracy of integration and subsequent wavelength-dependent corrections to the measured intensities). Firstly, the lattice parameters of the sample for a given experimental condition are not known precisely. It is also possible that, with an inaccurate orientation matrix, weak reflections (in regions of high or structured backgrounds) found by the peak-search routines may not be indexed by the determined orientation matrix.

It is possible that inaccuracy in the ability of the orientation matrix to index reflections may be a result of the inaccuracy of the description or model of the instrument itself within the software used to map the reflections onto the detector surface, and this can also lead to errors in the integrated peak intensities. For full data reduction and reliable intensities it is necessary to measure or calculate accurately the wavelength dependencies of many factors, including incident-beam spectrum detector efficiencies, and absorption and extinction corrections which are dependent on the Bragg angle (Schultz, 1993 ▶). Thus it can be seen that, with an improved description of the instrument, more reliable intensities can also be determined for reflections that occur in TOF spectra with a structured background, also permitting improved implementation of wavelength correction factors.

In this paper, we describe a new method of calibrating single-crystal TOF Laue neutron instruments, which can be used for many instruments at large-scale facilities. The new method uses a comprehensive mathematical model of the instrument and provides the user with accurate three-dimensional coordinates of all the instrument pixels, and hence 2θ values for the diffraction peaks. The detector positions are determined using neutron measurements, since an optical survey of the detector pixel locations is impractical in many single-crystal TOF instruments. The results of the neutron calibration measurements are combined using new software which has been written to calculate the parameters of the mathematical model together with their uncertainties. Having a good knowledge of the instrument detector positions relative to a given frame of reference will significantly improve the ability to determine lattice parameters, as the instrument description will remain fixed and only the crystal orientation and lattice parameters need be refined. It will also allow an increased precision in the ability of the orientation matrix to describe the crystal, and hence a more accurate determination of the reflection position on the detector surface. To date, the new method has led to a reduction in the uncertainty in the lattice parameters determined using the SXD instrument at the ISIS neutron source, and it has been shown that a cubic lattice parameter can be determined to 0.014% when 82 reflections are recorded. In the following sections of the paper we will describe why there is a need for improved descriptions of the orientation matrix and the instrument. We then describe the instrument for which this initial model was built and the mathematical model. We then describe the calibration procedure itself and finally the improvements the method has made in determining orientation matrices and instrument geometries.

## 2. The need for improved accuracy   

For most neutron single-crystal experiments there is a need to index reflections accurately and extract their intensities reliably, and this is particularly the case when studying incommensurate (Ling *et al.*, 2009 ▶) or magnetic structures (Agrestini *et al.*, 2008 ▶), or following phase transitions and surveying reciprocal space, for which an accurate map is required.

A recent example showing the need for accurate lattice parameters is the case of high-pressure single-crystal experiments (Bull, Guthrie, Nelmes, Loveday, Komatsu *et al.*, 2009 ▶), where it may only be possible to determine the pressure a sample is at from the unit-cell volume using a previously determined equation of state. However, for the sample pressure to be determined to a precision of 5% (for a sample with a bulk modulus of 24 GPa), the cell dimensions must be determined to better than 0.06% and, to date, Laue TOF single-crystal instruments have not been able to determine lattice parameters to the required degree of accuracy.

We therefore concluded that, in more complex single-crystal experiments, as described above, where weak reflections are present within the intrinsic background or more accurate determination of orientation matrices is required, it is vital to make maximum use of the limited information available by establishing the incoming and diffracted neutron trajectories precisely, along with the recorded TOF. This requires a complete understanding of the instrument collimation and detector geometry, and this has been established through the construction of a complete mathematical model of the instrument and its calibration by careful measurements.

## 3. The SXD instrument and software   

**Figure 1:**
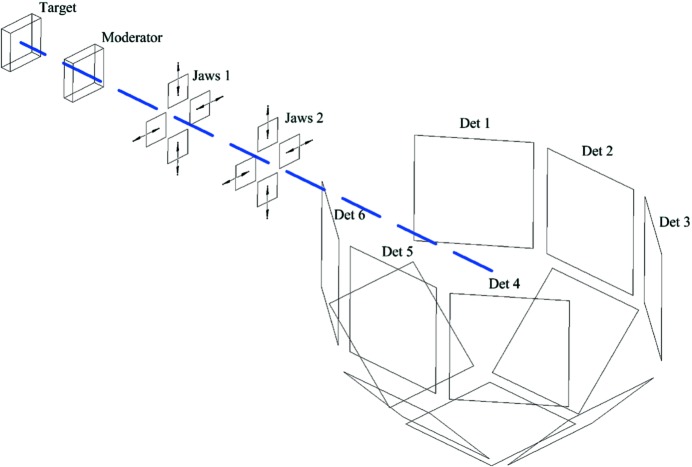
A schematic diagram of the SXD instrument. The neutrons leave the target and are energy-shifted by the water moderator. The size of the beam impinging on the sample position is defined by the adjustable BN jaws. The sample position is surrounded by 11 detectors, six in the equatorial plane and five below, maximizing the volume of reciprocal space that can be measured with one sample orientation. The frame that supports the detectors is shown in more detail in Fig. 2 ▶. Using the ISIS in-house software (*SXD2001*), the centre of each detector is defined by angles from the incident beam direction, denoted γ in the horizontal plane and ν_M_ in the vertical plane.

We begin with a description of the SXD instrument (on which the new calibration methodology has been initially tested) and the TOF Laue technique, which has been well described elsewhere (Keen *et al.*, 2006 ▶; Gutmann *et al.*, 2003 ▶). The SXD instrument (shown schematically in Fig. 1 ▶) is positioned 8.3 m from a Gd-poisoned ambient-temperature water moderator on Target Station One of ISIS. The sample position is surrounded by 11 ZnS scintillator detectors, each with an array of 64 × 64 3 mm^2^ pixels. The TOF Laue method means that a large number of reflections can simultaneously satisfy the diffraction condition, and hence a large volume of reciprocal space can be observed with a limited number of sample orientations.

The in-house ISIS software *SXD2001* (Gutmann, 2005 ▶) was written using the language IDL. This software has an idealized starting model of the SXD instrument using engineering coordinates. The adjustable parameters within the *SXD2001* model include three positions and two orientation angles for each detector, together with the sample position relative to the instrument, the orientation matrix and a TOF offset parameter; these are described by Keen *et al.* (2006 ▶). When the six equatorial detectors are used (as is the case for high-pressure studies), this makes a total of 43 parameters.

First, the Laue TOF spectra for each detector pixel are searched to find reflections from the sample. Reflections are located using a search algorithm, and the centre-of-mass positions of these reflections on the detector surface (*i.e.* two-dimensional pixel coordinates) and in time (within the TOF spectrum) are determined. These values are used to obtain an initial orientation matrix with corresponding lattice parameters. The orientation matrix, lattice parameters, sample position and instrument parameters are subsequently refined using a least-squares routine. The determined parameters are used to index all observed reflections found through the peak-search routines. It is then possible to integrate each reflection, correcting for wavelength effects and detector efficiencies and providing a value of *F* ^2^, a position in *x*, *z* and a TOF for each observed reflection which fit the determined orientation matrix in a given *hkl* tolerance.

## 4. The SXD frame of reference   

**Figure 2:**
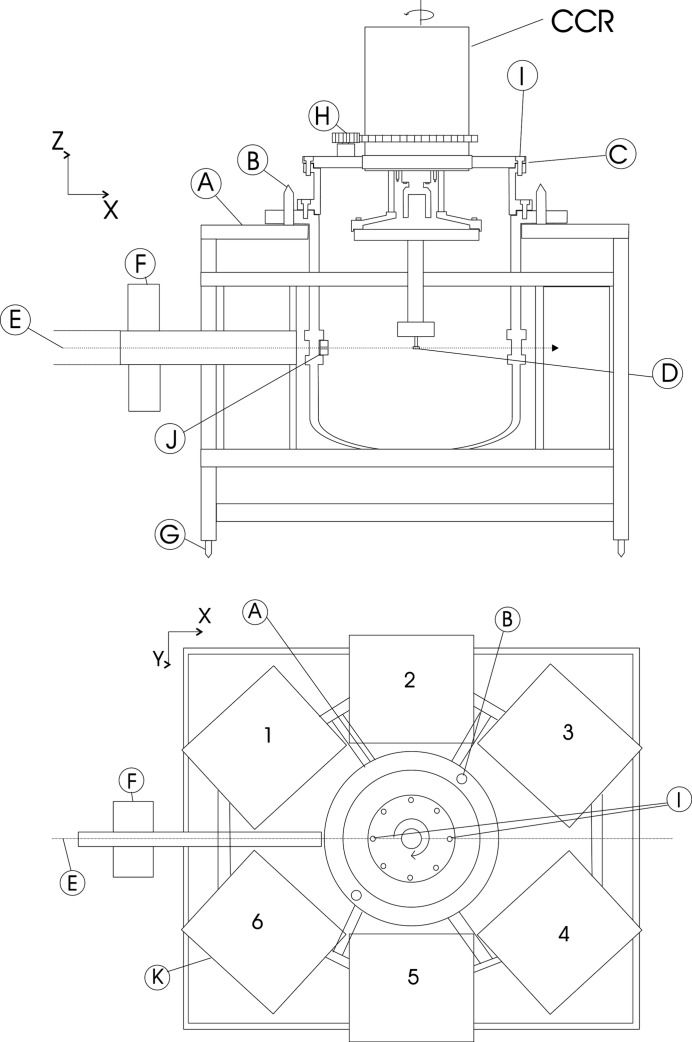
(Not to common scale) (Top) A schematic diagram of the frame of reference defining the **z** direction. (Bottom) A schematic diagram of the frame of reference defining the **x** and **y** directions. A is the frame of SXD upon which are mounted the detectors and the CCR. Upon the SXD frame are two dowel pins labelled B onto which are located the tank of the CCR. From a datum point which is the surface labelled C, a position 300 mm below this is defined as the sample position D. A perpendicular line from surface C defines the **z** direction of the frame of reference. E is the suggested incident beam direction of SXD, F are the jaws defining the SXD beam size, G are kinematic mounts defining the SXD frame relative to the moderator and target and hence E, H is the rotation mechanism which rotates the crystal around the origin of the axis, and I are pins used to define the **x** direction, which is defined to pass through a line that can be drawn through these two points. The **y** direction is defined as being perpendicular to a plane which is parallel to that defined by the datum surface labelled C. J is additional beam-defining collimation.

A prerequisite for establishing a reliable model of the instrument is to define a suitable frame of reference. Such a frame should permit the sample to be placed reproducibly at the same location and enable a set of axes to be defined. For the model of SXD, we have used its own mechanical frame and specialized sample-mounting equipment described previously (Bull, Guthrie, Nelmes, Loveday, Komatsu *et al.*, 2009 ▶; Bull *et al.*, 2009 ▶). This frame of reference is shown schematically in Fig. 2 ▶, and the method described here assumes that the relative positions and orientations of all the detectors and the sample position are fixed by the SXD frame (labelled A in Fig. 2 ▶).

The fundamental point of reference is the top ring, which holds the sample mount in a fixed reproducible position and orientation with respect to the frame (labelled I). By rotation around this ring, we define an axis, and the reproducible sample position is defined as the point on that axis that is 300 mm below the ring (labelled C). This position is the origin of the *x*, *y* and *z* axes. The rotation axis is defined as the *z* axis.

Fiducial marks in a top plate set in the support ring define a direction labelled I, and the *x* axis is in the direction parallel to that and passing through the reference sample position.

The reference frame is completely defined by its origin, the two axes (*z*, *x*), *y* being such as to form a right-handed set of axes with a vertical *z* axis and *x* increasing in the neutron beam direction.

## 5. The new mathematical model of SXD   

The new programs *SXDCALIB* and *SXDMEASURE* (Johnson *et al.*, 2013 ▶) (§6) incorporate a mathematical model to define the positions of all the detector pixels relative to the frame of reference described above. For the SXD instrument at ISIS, the model currently defines the positions of each of the 4096 pixels of each of the six SXD equatorial detectors. To do this we first define the pixel position within the coordinate system of each detector and then calculate this position in the instrument coordinate system.

**Figure 3:**
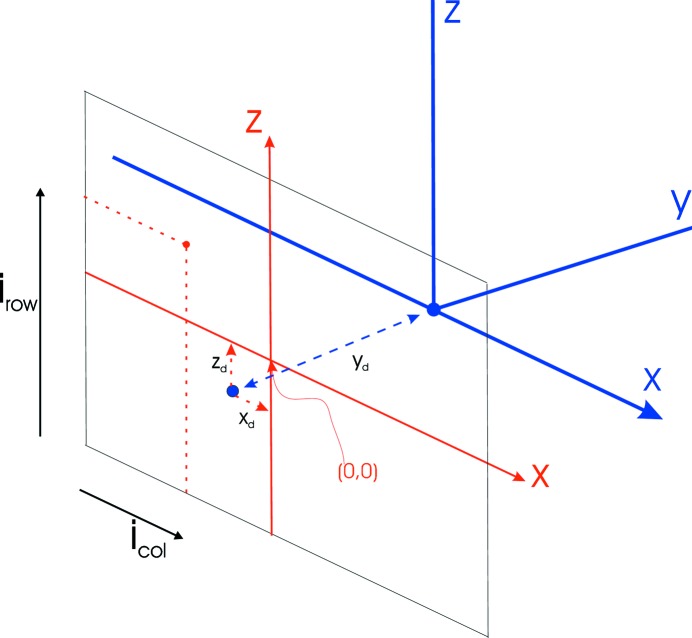
Detector 5, showing the pixel positions within the detector coordinate system (red) and the instrument coordinate system (blue).

Fig. 3 ▶ illustrates a single detector with its coordinate system (shown in red) and the instrument coordinate system (shown in blue). The ‘*y*’ axis is defined to be perpendicular to the ‘*xz*’ plane, the ‘*x*’ and ‘*z*’ directions having been defined above (§4).

Each detector is modelled as a planar object consisting of regularly placed pixels lying on a square 64 × 64 array. The numbering convention used to define the pixel positions on the detector *xz* plane is shown in Fig. 3 ▶, where the detector is viewed from the back. Pixel, row and column numbers start from the bottom left, and pixel numbers (which run from 1 to 4096) increase up each column in turn.

Thus, the pixel number *k* for a pixel in row *i*_row_ and column *i*_col_ is
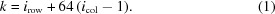
We define the distance between pixels as px, pz and the centre of the detector has detector coordinates of (0, 0). Thus, in the detector coordinates a pixel at position (*i*_col_, *i*_row_) will lie at
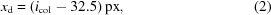


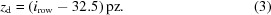
To determine the position of each pixel in the instrument coordinates, the detector must be moved to its correct position in space. Each detector has six coordinates, three defining the centre of the detector (*x*_d_, *y*_d_, *z*_d_) and three rotation angles (φ_*x*_, φ_*y*_, φ_*z*_), permitting the detector to occupy any position in space. The starting position of the detector is with its centre at (0, 0) (in the instrument coordinates), with the detector *x* and *z* axes coincident with those of the instrument frame. The orientation is such that the front of the detector faces the positive **y** direction. Hence, pixel 1 has negative *x* and *z* values. The detector reaches its final position by first rotating through an angle of φ_*x*_ (about the *x* axis), then φ_*y*_ (about the *y* axis) and finally φ_*z*_ (about the *z* axis). The detector centre is then moved to the location (*x*_d_, *y*_d_, *z*_d_). Thus, the detector shown in Fig. 3 ▶ is an idealized detector 5, with φ_*x*_ = φ_*y*_ = φ_*z*_ = 0, and with the detector centre lying at the instrument coordinates (*x*_d_, *y*_d_, *z*_d_). In this simplified case the instrument coordinates at which the pixel (*i*_col_, *i*_row_) will lie are
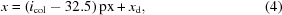


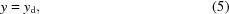


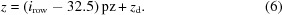
Since all the detectors calibrated in the current work are nominally vertical and lie in the equatorial plane, the values of *y*_d_, φ_*x*_ and φ_*y*_ will be small. The other parameters in the model of the instrument include the primary flight path *L*_0_ (from the moderator to the sample), the direction cosines of the incident neutron beam to the *x* axis (α_n_, β_n_), the position of the sample (*x*_s_, *y*_s_, *z*_s_), the orientation of the single crystal (ϕ_c_, φ_c_, γ_c_) and its lattice parameters (*a*, *b*, *c*, α, β, γ). Thus, the model includes a potential maximum of 63 parameters (compared with 43 for the existing description of the SXD instrument).

## 6. Calibrating SXD   

Having established a reproducible reference frame on the physical instrument, the next step was to relate this to the mathematical model. Because the mathematical model contains a number of parameters that are highly correlated, it was important to fix some of the parameters from independent measurements before a final single-crystal calibration was undertaken. The parameters were therefore fixed in the following order.

(*a*) The mean pixel size (px) was readily determined from metrology of the physical structure of the detector. The CNC/photo-etch process used to construct the SXD pixel spacing is known to deliver a very reproducible inter-pixel spacing, and the precise value of this parameter was measured to be 3.000 (1) mm. This value was also confirmed by the powder diffraction results (§6.1).

(*b*) The *y* component of the neutron beam direction (α_n_) and the *x* position of detector 2 were determined from powder diffraction measurements (§6.1).

(*c*) The height of the centre point (*z*_d_) of each detector and its tilt (φ_*y*_) were determined from an ‘equatorial line’ determined from a series of aligned single-crystal measurements (§6.2).

(*d*) The remaining model parameters were evaluated from a refinement of KDP single-crystal data (§6.3).

### 6.1. Powder diffraction measurements   

A compacted rod of powdered nickel was placed at the defined instrument centre and data were collected for a sufficient time to record satisfactory powder diffraction patterns in all of the 24 576 pixels within the six SXD equatorial detectors. The nickel rod was then moved 70.0 (1) mm upstream from the defined instrument centre along the defined **x** direction and a powder diffraction pattern again obtained at each of the pixels of the detectors. A final diffraction pattern was obtained with the nickel rod displaced 70.0 (1) mm downstream from the defined instrument centre. Metrological observation methods were used to ensure that the nickel rod was centred to within 0.1 mm on the centre-of-instrument axis and that the displacements up- and downstream were made along the defined beam direction as defined by the frame of reference (see §4). The rod was shown by metrology to be on this defined line to within 4.5 mrad.

The resulting powder patterns were then normalized for detector efficiency on a pixel by pixel basis using the in-house software *SXD2001* (Gutmann, 2005 ▶), and output in a form suitable for the *GSAS* software package (Larson & Von Dreele, 1994 ▶).

The time of flight *t* in microseconds for a powder diffraction peak is given by the expression
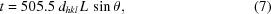
where *d*_*hkl*_ is the lattice spacing in ångström of the *hkl* reflection, 2θ is the angle of diffraction and *L* is the total flight path of the neutron in metres. The *GSAS* Rietveld refinement software was then used to calculate the quantity *L*sinθ (and its uncertainty) for each pixel using the known lattice parameter (and hence *d*_*hkl*_) for the nickel sample, which had been previously determined on the high-resolution powder diffraction (HRPD) instrument at ISIS (Ibberson *et al.*, 1992 ▶).

Since powder data are cylindrically symmetric, three-dimensional pixel positions cannot be determined, but with three separate values of *L*sinθ from three samples lying on the *x* axis it is possible to use a least-squares program to refine the *x* position of a row of pixels (together with φ_*y*_ and φ_*z*_) and the horizontal direction cosines (α_n_) of the incident neutron beam. A least-squares program *PIXPOS* was written to refine these parameters and the values obtained are listed in Table 1 ▶ (under origin ‘*b*’).

### 6.2. Definition of equatorial line   

A cube-shaped single crystal of KDP (KH_2_PO_4_, 99.99% from Cleveland Crystals, USA) of 27 mm^3^ in volume was mounted on a standard goniometer, suspended from the closed cycle refrigerator (CCR) described previously (Bull, Guthrie, Nelmes, Loveday, Komatsu *et al.*, 2009 ▶). The height of the sample was adjusted so that the centre of the crystal would be 300 mm below flange A as shown in Fig. 2 ▶. The crystal was then centred optically, so that when it was rotated about the vertical axis (**z** direction) the deviation was less than 0.1 mm from the axis, and it was shown that the maximum deviation in goniometer height for one full 360° rotation was ±0.045 mm.

The *SXD2001* software was used to determine the orientation matrix of the crystal (by the standard method as described previously) and to provide the measured location and integrated intensities for each reflection. The software calculates the *x*, *z* position of the reflection (in detector pixel coordinates), its time of flight (in microseconds) and the total number of neutrons recorded in the reflection. These values are determined by fitting a model of the peak (in *x*, *z* and *t*) to the observed neutron TOF spectra at adjoining pixels.

The position of each reflection was also determined by hand as a confirmation of the values determined by the three-dimensional fitting method used by *SXD2001*. A manual integration of each reflection, taking into account the detector efficiency, was made for each pixel containing a particular reflection (in a fixed resolution-determined TOF window) by fitting a Gaussian. The positions of the reflections were then determined by a weighting scheme based upon the numbering of the pixels and the integrated intensity within each of them. The difference between this manual method and the automated three-dimensional method was found to be less than 0.1 pixels and was checked for all detectors at random locations.

Using the determined pixel positions of the reflections, the crystal was then aligned with its *c* axis coincident with the rotation axis of the instrument (*z*). This was accomplished by recording the 440 reflection (or equivalents) at detectors 2 and 5 with the crystal rotated ±90° about the *z* axis.

By measuring the positions of the 440 set of reflections relative to each other, it was possible to adjust the arcs to bring opposite reflections into coincidence. After each adjustment of the arcs, the centring of the crystal on the rotation axis and its height were also checked. By performing this operation using both goniometer arcs, the crystal was aligned to the point where the centre of the 440 set of reflections observed when the arcs were 180° apart lay within 0.1 pixel (= 0.08°).

The mean location of opposite pairs of reflections accurately defines a plane at right angles to the rotation axis (ω) if the incident neutron beam is also normal to the rotation axis. We started by making this assumption and ultimately correcting for the small change this produces, as described in §6.3.

Diffraction data were collected at four orientations 90° apart in order to observe the 440 reflection and equivalents, so that all four reflections were observed within 0.1 pixels in the **z** direction. Data were collected with the crystal rotation ω (see Fig. 1 ▶) set to place these reflections at three positions across each detector, at *x* ≃ −22, 0 and 22 (pixels), a total of 36 orientations. The data were then analysed and integrated using *SXD2001* to determine the *z* coordinates of the 440 reflections on all detectors, and equatorial lines were drawn taking the averages of the *z* coordinates from different ω positions. The determined mean values were then used to define the height (*z* coordinate) of the centre of each detector, along with its tilt relative to the *xy* plane (φ_*y*_), and these values are shown in Table 1 ▶ (under origin ‘*c*’).

### 6.3. Single-crystal calibration measurements   

The KDP single-crystal data sets described in §6.2 also provided out-of-plane reflections. These complete data sets were analysed using *SXD2001* to yield sets of {*hkl*, *x* (in pixels), *z* (in pixels), TOF, *I*} values. Each of the 36 orientations of the KDP crystal provided around 260 reflections.

One of these data sets, containing 282 reflections, was then used as input to the least-squares program *SXDCALIB* (Johnson *et al.*, 2013 ▶) to refine the remaining SXD instrumental parameters. *SXDCALIB* is one of a pair of least-squares programs (*SXDMEASURE* being the other) which share common code. *SXDCALIB* enables the user to refine the instrument parameters using reflections from a crystal with known lattice parameters. *SXDMEASURE* uses the same mathematical model to refine the lattice parameters of an unknown crystal while keeping the instrument parameters fixed. They both calculate the mismatch between the observed SXD data {*x* (*hkl*), *z* (*hkl*), TOF (*hkl*)} and values calculated from a knowledge of the instrument dimensions, crystal lattice parameters and crystal orientation (orientation matrix). The values of the lattice parameters for the tetragonal phase of KDP used in this calibration were *a* = 7.4529 (2) and *c* = 6.974 (2) Å at 298 K (Cook, 1967 ▶). The program employs a simple algorithm (multiple linear minimization) for determining the minimum value of a multidimensional function, described in *Numerical Recipes* (Press *et al.*, 1989 ▶).

The uncertainties of each of the refined parameters are determined by the robust Monte Carlo method of repeating the calculation with new data sets, derived from the original by ‘dressing’ each of the data (and fixed parameter) values with an error, correctly sampled according to the uncertainty assigned to the data. Thus, as well as handling the uncertainties of the reflection data correctly, the *SXDCALIB*/*SXDMEASURE* programs also enable users to specify the uncertainties of the ‘fixed’ parameters used in a refinement.

The least-squares procedure minimizes the quantity *S* (for all reflections 1 → *n*):
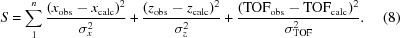
From metrology, powder diffraction refinements and equatorial line measurement, we have determined the px, *x*_d*i*_, *z*_d*i*_ and φ_*i*_ coordinates in our model and, combining these with the data sets of *h, k, l, x* (in pixels), *z* (in pixels) and TOF (in microseconds) (and their known uncertainties), it is possible to obtain the full detector parameter set (and their uncertainties) using *SXDCALIB*. Table 1 ▶ lists the final refined parameters from the single-crystal refinements (under origin ‘*d*’).

It should be noted that the detector *z* coordinates determined from the single-crystal ‘equatorial line’ measurement are only correct if the neutron beam is exactly at right angles to the *z* axis (*i.e.* β = 0). Since this is not necessarily true, the least-squares refinement includes an appropriate adjustment to the detector height which is determined from the value of β.

The result of the KDP refinement was a χ^2^ value of 2.9, and average values for the errors of the reflection positions of 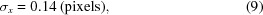


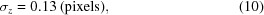


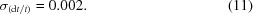
One important aspect of the analysis revealed that it is important to use exactly the same methodology to calibrate the instrument as is used to determine the orientation matrix of the sample. In particular, the standard *SXD2001* software can determine the three reflection coordinates [*x* (in pixels), *z* (in pixels) and *t* (in microseconds)] in two ways depending upon the type of peak-fitting procedure used. One set, called the ‘USE’ data file, is based upon the initial simple and fast peak-search algorithm. The other, the ‘3D’ data file, is determined from the more accurate but slower integration of the reflections in which the pixel and TOF positions are fitted taking into account the TOF asymmetry. Thus, if a ‘3D’ KDP data set is used in the calibration of SXD instrument parameters, a ‘3D’ data set should be used from the crystal for which the unknown lattice parameters are to be determined. The values shown in Table 1 ▶ are from ‘3D’ data.

## 7. Determining lattice parameters from the calibrated detector positions   

### 7.1. Results from refined lattice parameters   

With a full set of detector parameters {*x*_d*i*_, *y*_*d*i_, *z*_*d*i_, ϕ_*i*_, φ_*i*_, γ_*i*_, px, *L*_0_, α_beam_, β_beam_}, the correct location of any measured diffraction peak may now be determined in the instrument coordinate system. This location provides an accurate 2θ value for the reflection which, together with its TOF value, may be used to calculate the crystal orientation and its lattice parameters.

The program *SXDMEASURE* was used to obtain the lattice parameter(s) of a new sample, using the detector parameters determined by the KDP refinement as fixed parameters within the refinement, with the refined parameters being the sample orientation and its lattice parameter(s). To check this new method, diffraction data were taken from two single crystals with known lattice parameters. The first sample was a cylinder of sodium chloride (NaCl, 12 mm^3^ in volume, 99.99% pure obtained from *CRYSTAN*, UK, and kept in an anhydrous environment), mounted at the 300 mm position below frame A (Fig. 2 ▶) and shown to be on the rotation axis and hence at the instrument centre to within 0.1 mm. A diffraction pattern was collected for a period of 2 h at a random orientation. The reflections were indexed using *SXD2001*, using an initial value for the NaCl (cubic) lattice parameter of 5.6402 Å. The resulting data sets of {*h*, *k*, *l*, *x* (in pixels), *z* (in pixels), TOF (in microseconds)} were then used as input to *SXDMEASURE*, with the predetermined detector parameters {*x*_d*i*_, *y*_d*i*_, *z*_d*i*_, ϕ_*i*_, φ_*i*_, γ_*i*_, px, *L*_0_, α_beam_, β_beam_} held constant as described in §6.

*SXDMEASURE* returned a value for the NaCl lattice parameter of *a* = 5.6403 (8) Å. The refinement used a total of 90 NaCl reflections and reached a final χ^2^ value of 1.9. The errors in the reflection positions are detailed in Table 2 ▶.

This demonstrates that the method can calculate a single lattice parameter to an uncertainty in the refined value of 0.014%. The value we have calculated using this method is also within 1σ of the published value for *a*_NaCl_ [5.6402 (2) Å; Linde, 1991 ▶].

The second sample was the monoclinic crystal spodumene [LiAl(SiO_3_)_2_] from Black Hills, Dakota, USA. Electron microprobe analysis showed no elemental impurities. Following a similar procedure, the data from a single orientation were used to refine the four lattice parameters of the crystal. The refinement used a total of 328 reflections and reached a final χ^2^ value of 1.7, and refined values for the spodumene lattice parameters are detailed in Table 2 ▶.

The lattice parameter uncertainties in this example lie in the range 0.02–0.05%. The values for *a, b, c* and β also agree closely with those determined using the HRPD instrument, with a root mean-square (r.m.s.) difference of 1.3σ.

When the number of reflections in the data set is restricted, to simulate the effect of a crystal being inside a pressure cell or other complex sample environment (and hence reduced access to reciprocal space and data coverage), the uncertainty in the final lattice parameter determination will naturally increase. To test this effect, the spodumene data were reduced to 128 reflections by using only those found in a window of ±10° from the detector centre, and this gave comparable results as shown in Table 2 ▶. The lattice parameter uncertainties in this example lie in the range 0.02–0.06%. The values for *a, b, c* and β also agree with those determined using the HRPD instrument, with an r.m.s. difference of 1.5σ (see above). In addition to providing accurate values of the lattice parameters (we note the *a* axis is affected the most, but as the crystal was oriented in this sense it is not surprising), *SXDMEASURE* determines an orientation matrix which can then be used (in conjunction with the instrument description) to provide good predictions of the reflection positions on each detector as detailed in Table 2 ▶, even with the restricted access to data.

### 7.2. Wavelength range used in data sets   

As noted above, it has become apparent that it is critically important when determining lattice parameters that the same procedure is used to calibrate the detector positions as is used to determine the lattice parameters of a new sample. This principle may be called the principle of ‘least difference’. That is, when measuring a new sample the same procedure should be used as that used to calibrate the instrument, and using the same wavelength range within the refinements.

The need to use data with similar wavelength ranges became apparent when initially studying the results of the NaCl *a* lattice parameter determined from different detectors. It was noticed that, if data from different pairs of detectors (*e.g.* 1 and 6, 2 and 5, 3 and 4) were used in the refinement, a systematic shift (of about 0.1%) in the value of *a*_NaCl_ was observed. On further investigation it was noted that this corresponded to the different wavelength ranges recorded by each pair of detectors. It is therefore very likely that the wavelength range employed introduces a subtle systematic bias to the outcome of a refinement. This could well be a result of the significant changes in the resolution function as the wavelength and angle change around the instrument (where, for a given wavelength, upon increasing the 2θ angle the intrinsic spatial resolution increases).

To mitigate this effect, the methodology employed here was to calculate the average TOF used across all six detectors in the KDP calibration refinement and then remove reflections from the ‘unknown’ data set, to bring the average TOF of the two data sets into close agreement. This always involved removing reflections with low TOF values, and most of these were recorded in the low-angle detectors 3 and 4, where the peak positions are clearly more difficult to determine because of the lower resolution. The mean TOF of the KDP calibration was 2614 µs.

Using this ‘matched’ TOF approach, the excellent results shown above were obtained. The approach is also clearly part of the least-difference approach to making accurate measurements. However, for the actual structure refinement, all reflections observed are used for the final *hkl, F* ^2^ data set.

### 7.3. Existing SXD methods for lattice parameter determination   

Since this paper puts forward a new method for lattice parameter determination, this section presents a comparison between the new method (*SXDMEASURE*) and previous SXD software. The *SXD2001* program uses a more limited model of the SXD instrument (see §3), and to provide a complete comparison we present the results obtained in three ways:

(*a*) Using the SXD model with the detector positions and beamline parameters held fixed at their nominal engineering values. This is marked ‘As engineered SXD’ in Table 2 ▶.

(*b*) Using the SXD model but allowing the detector positions to refine along with the lattice parameters and orientation of the crystal under study. This is marked as the ‘Standard’ method in Table 2 ▶.

(*c*) Calibrating the SXD model using the KDP data in a manner analogous to that used for the *SXDMEASURE* model. This is marked as ‘Calibrated’ in Table 2 ▶.

For method (*c*), the lattice parameters of KDP were fixed to their known values and only the parameters describing the detectors and crystal orientation were allowed to vary. The detector parameters were subsequently stored as a file that describes the instrument. The parameters themselves only vary by a small amount from the engineered values, as shown in Table 1 ▶. These instrument parameters were fixed and then used to refine the lattice parameters and orientation matrices for the data collected from the NaCl and spodumene single crystals described above. The results of these refinements are shown in Table 2 ▶. When we started work on the *SXDMEASURE* method, only methods (*a*) and (*b*) were in use on SXD.

We have also measured the ability of the refined orientation matrix for each method to predict the position and time each reflection is predicted to appear in the SXD instrument, and the errors are shown in Table 2 ▶ as σ_*x*_, σ_*z*_ and σ_d*t*/*t*_. An extension of this is to look at the number of reflections in the whole data set for which accurate TOF predictions can be made within a given value, and this is described in Table 3 ▶.

## 8. Discussion   

The method described above has been shown to provide an accurate technique for refining unknown lattice parameters on the SXD instrument at ISIS.

In Table 1 ▶ we give the refined and nominal values of the parameters used to describe the SXD detectors for the *SXDMEASURE* model. These parameters are used in the mathematical model of SXD, which is used to determine the lattice parameters and orientation matrices of crystals placed at the defined instrument centre. The changes in the parameters from the nominal engineering values themselves are quite small. However, as can be seen in the improvement in the accuracy and precision of the determined lattice parameters, they are a significant factor in improving the measurement of the crystal parameters.

The small changes in the parameters used to describe the SXD instrument by the *SXD2001* software (when this model is calibrated) are also observed to improve the refined lattice parameters. For example, if the detector positions are set to their engineering values (and not refined), the lattice parameters determined for NaCl and spodumene differ from their known values by between 0.1 and 1%. Using a calibrated *SXD2001* model improves this considerably, and the lowest uncertainties are obtained using the *SXDMEASURE* procedure.

It is important to note that, using the uncalibrated engineering values for the instrument parameters, the errors of the calculated reflection positions are much higher than when calibrated methods are used (Table 2 ▶). A similar trend in the ability to predict the TOF for a given reflection is also shown in Table 2 ▶ as the values of σ_d*t*/*t*_ decrease with increasing complexity of the model used to describe the SXD instrument. The accuracy in the lattice parameter measurement (0.014% for a cubic crystal) is much improved over the ‘standard SXD’ lattice parameter refinement technique, which does not use a calibration refinement to determine the detector positions.

This leads us to discuss the way in which the new model and calibration of the SXD instrument can be used. For a user wishing to improve on the determined lattice parameters there are two methods that can be used. Firstly, there is the use of the existing in-house *SXD2001* software and implementation of the new calibration information. This already dramatically improves the determination of the lattice parameters (providing the sample is placed at the defined instrument centre). For further improved accuracy, the values of the peak positions and TOF can be obtained by integrating the data using the *SXD2001* software (‘3D’ data) and using these values in the *SXDMEASURE* program. As noted above, it is important to match the average TOF of the measured reflections to that used in the calibration (2614 µs) to ensure the most accurate results.

For a typical user of SXD the first question is, What are the orientation of the crystal and the lattice parameters? If the experiment allows access to a large region of reciprocal space which is well populated with reflections, this can be readily achieved.

It is common that, during the first period of data collection, reflections are first observed in the low-angle detector banks (detectors 3 and 4; Fig. 1 ▶). However, such reflections tend to be less well defined (in both TOF and pixel position) than those recorded in the 90° detector banks (2 and 5). It is these reflections which more accurately determine the orientation matrix and this information can then be used to guide further data-collection strategies.

With longer data-collection times, high-resolution reflections can be observed in the high-angle banks (1 and 6) in the longer-wavelength range. However, in the high-angle banks the background increases with increasing θ and also the neutron flux decreases, making reflections inherently weaker. This is where, with improved data sets, the improved SXD calibration can be used and the indexing and location determination of weaker reflections at high *Q* in the high-angle detector banks can be improved.

For a user with reduced access to reciprocal space, a weakly scattering sample or a limited number of reflections, the above methodology must be changed, and being able to use the low-angle banks reliably becomes more important. Hence, using the calibrated SXD instrument parameters becomes significant. This is also critical for experiments where a certain sample condition is required (*i.e.* lattice parameter) which is tuned *in situ* by applied temperature, pressure, or electric or magnetic field. It is necessary to know that the correct sample conditions are met in a reasonably short data-collection time (given the cost and scarcity of beamtime at a large-scale facility), and hence rapid and reliable lattice parameter determination is critical.

It is also useful to know how well the orientation matrix can predict reflection positions. We therefore compare the ability of an orientation matrix determined only from data collected at detectors 3 and 4 to predict positions and TOF for reflections in detectors 1, 2, 5 and 6. As can be seen in Table 4 ▶, significant improvement is made by using the more complete descriptions of the SXD instrument, and *SXDMEASURE* was able to predict almost all reflection positions within half a pixel, compared with less than 20% using the other techniques.

The calibration method and associated software described in this paper can be used on other single-crystal instruments at pulsed neutron sources. The description of the detector geometry could be readily incorporated into the two programs *PIXOS* and *SXDMEASURE*, which are written in Fortran. These programs could also be transferred to the *MANTID* suite of programs for data visualization and reduction, which is being used regularly at spallation neutron sources worldwide (Taylor *et al.*, 2012 ▶).

The improvements over existing techniques for TOF single-crystal instrument calibration are a result of the improved description of the detector geometry, the use of the least-difference principle and the ability of the software to calculate correctly the errors of the measured values.

## Figures and Tables

**Table 1:** A list of all the SXD model parameters, together with their uncertainties, nominal engineering values, origin and units The origins of the parameter values were (*a*) metrology, (*b*) powder measurements, (*c*) ‘equatorial line’ determination and (*d*) refinement of known KDP single-crystal reflection data (see §6).

Parameter	Model value	Nominal value	Origin/units	Parameter	Model value	Nominal value	Origin/units
px, pz	3.000 (1)	3.0	*a*/mm	α_n_	0.00043 (2)	0.0	*b*/rad
*L*_0_	8297.3 (6)	8300	*d*/mm	β_n_	−0.0003 (4)	0.0	*d*/rad
*x*_d1_	−176.1 (2)	−178.5	*d*/mm	*x*_d4_	177.29 (20)	178.5	*d*/mm
*y*_d1_	139.43 (14)	136.98	*d*/mm	*y*_d4_	−138.79 (19)	−136.98	*d*/mm
*z*_d1_	−0.73 (9)	0.0	*c*/mm	*z*_d4_	−2.04 (8)	0.0	*c*/mm
φ_*x*1_	0.0161 (30)	0.0	*d*/rad	φ_*x*4_	0.0034 (39)	0.0	*d*/rad
φ_*y*1_	0.0023 (11)	0.0	*c*/rad	φ_*y*4_	−0.0027 (17)	0.07	*c*/rad
φ_*z*1_	4.0522 (24)	4.0369	*d*/rad	φ_*z*4_	0.8951 (31)	0.9163	*d*/rad
*x*_d2_	−1.41 (1)	0.0	*b*/mm	*x*_d5_	−3.12 (14)	0.0	*d*/mm
*y*_d2_	226.5 (2)	225	*d*/mm	*y*_d5_	−225.2 (2)	−225	*d*/mm
*z*_d2_	−0.13 (10)	0.0	*c*/mm	*z*_d5_	−1.39 (12)	0.0	*c*/mm
φ_*x*2_	0.0073 (40)	0.0	*d*/rad	φ_*x*5_	0.0190 (50)	0.0	*d*/rad
φ_*y*2_	0.0004 (10)	0.0	*c*/rad	φ_*y*5_	−0.0032 (10)	0.0	*c*/rad
φ_*z*2_	3.105 (48)	3.1416	*d*/rad	φ_*z*5_	0.0028 (31)	0.0	*d*/rad
*x*_d3_	178.15 (23)	178.5	*d*/mm	*x*_d6_	−177.95 (20)	−178.5	*d*/mm
*y*_d3_	138.49 (17)	136.98	*d*/mm	*y*_d6_	−138.14 (13)	−136.98	*d*/mm
*z*_d3_	−0.51 (11)	0.0	*c*/mm	*z*_d6_	−2.02 (10)	0.0	*c*/mm
φ_*x*3_	0.0187 (33)	0.0	*d*/rad	φ_*x*6_	0.0127 (36)	0.0	*d*/rad
φ_*y*3_	0.0038 (20)	0.0	*c*/rad	φ_*y*6_	−0.0092 (11)	0.0	*c*/rad
φ_*z*3_	2.2322 (36)	2.2253	*d*/rad	φ_*z*6_	−0.9098 (27)	−0.9163	*d*/rad

**Table 2:** Lattice parameters determined using the methods described within this paper The values determined from cubic NaCl and monoclinic spodumene are shown. These values were determined both as a standard user of the existing understanding of the SXD instrument would determine them and using the new calibration of the instrument described in this paper. The results from the new *SXDMEASURE* method are shown and compared with the values determined using the HRPD instrument. Finally, a comparison is made for spodumene, where a restricted data set is used which restricts access to reciprocal space to ±10° from the horizontal direction on the detector surface. Where no value is given no value was refined.

Data set	*a* (Å)	*b* (Å)	*c* (Å)	β (°)	σ_*x*_ (pixels)	σ_*z*_ (pixels)	σ_d*t*/*t*_
NaCl (90 reflections)							
As engineered SXD	5.6554 (59)	–	–	–	0.75	0.35	0.0044
Standard	5.6519 (57)	–	–	–	0.15	0.17	0.0020
Calibrated	5.6394 (55)	–	–	–	0.16	0.17	0.0019
*SXDMEASURE*	5.6403 (8)	–	–	–	0.15	0.13	0.0013

Spodumene (328 reflections)							
As engineered SXD	9.480 (9)	8.476 (9)	5.231 (4)	110.28 (4)	0.71	0.32	0.0037
Standard	9.479 (16)	8.429 (14)	5.232 (5)	110.20 (8)	0.15	0.19	0.0012
Calibrated	9.471 (9)	8.394 (9)	5.220 (4)	110.21 (4)	0.15	0.19	0.0019
*SXDMEASURE*	9.459 (3)	8.397 (4)	5.218 (1)	110.17 (3)	0.14	0.14	0.0011
HRPD	9.46440 (16)	8.38986 (6)	5.21866 (42)	110.1747 (12)			

Restricted data set of spodumene (128 reflections in ±10°)							
*SXDMEASURE*	9.476 (6)	8.397 (5)	5.218 (1)	110.21 (2)	0.14	0.18	0.0012

**Table 3:** Demonstration of how the ability of an orientation matrix to predict the TOF for a given reflection is an important criterion in determining its accuracy Here, the percentage of reflections for which the orientation matrix can predict the correct TOF within a given tolerance is shown, and it can be seen that, with increasing instrument description complexity, the ability to predict TOF correctly increases. d*t*/*t* is defined as the difference between the observed and calculated TOF for a given reflection (d*t*) divided by the TOF for the reflection.

Calibration method	|d*t*/*t*| ≤ 0.001 (%)	|d*t*/*t*| ≤ 0.002 (%)	|d*t*/*t*| ≤ 0.005 (%)
As engineered	33	54	81
SXD normal	45	74	94
SXD calibrated	52	84	98
*SXDMEASURE*	67	90	99

**Table 4:** A comparison of the ability of an orientation matrix to predict the position and time of reflections at detectors 1, 2 and 5 The orientation matrix was determined from data collected only at detectors 3 and 4 (as would happen at the beginning of a data collection) and used to predict reflection positions at detectors 1, 2, 5 and 6. Note that the normal SXD method does not allow calibration of the detector parameters at detectors 3 and 4 and hence is not included in this comparison. Here, d*r* is defined as d*r* = (d*x* ^2^ + d*z* ^2^)^1/2^, where d*x* and d*z* are the difference between the observed and calculated reflection positions on the detector surface. *I* is defined as 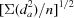.

Calibration method	d*r* < 0.2 pixels (%)	d*r* < 0.3 pixels (%)	d*r* < 0.5 pixels (%)	σ_*x*_ (pixels)	σ_*z*_ (pixels)	σ_d*t*/*t*_
As engineered	0.94	5.7	10.3	0.76	0.44	0.0055
SXD calibrated	3.8	5.7	18.9	0.47	0.59	0.056
*SXDMEASURE*	18	50	97	0.13	0.15	0.0009
